# Changes of corneal tomography in patients with congenital blepharoptosis

**DOI:** 10.1038/s41598-017-06823-7

**Published:** 2017-07-26

**Authors:** Tiepei Zhu, Xin Ye, Peifang Xu, Jingyi Wang, Huina Zhang, Hailong Ni, Zhaoan Su, Juan Ye

**Affiliations:** Eye Center, The Second Affiliated Hospital, School of Medicine, Zhejiang University, Zhejiang, China

## Abstract

The study aimed to evaluate the effect of drooped eyelid on corneal tomography in congenital blepharoptosis patients. Sixty-four patients with congenital blepharoptosis and 64 age- and sex- matched healthy subjects were included. According to the eyelid margin to corneal light reflex distance (MRD), eyes with congenital blepharoptosis were categorized as mild, moderate, or severe. The eyes were scanned using the rotating Scheimpflug camera. Increased topometric parameters were observed in moderate and severe blepharoptosis. Back corneal elevations at the thinnest point were significant higher for mild (*P* = 0.009), moderate (*P* < 0.001), and severe (*P* < 0.001) congenital blepharoptosis compared with controls. Maximum Ambrósio’s relational thickness (ART) was decreased in eyes with severe blepharoptosis (*P* < 0.001). Fnal D values were significantly higher in moderate (*P* < 0.001) and severe blepharoptosis (*P* < 0.001) groups than that of controls. There were significant correlations between MRD and most corneal tomographic parameters. Our findings indicated there was a trend toward subclinical keratoconus-like changes in the corneas of congenital blepharoptosis, with the increase of ptosis severity.

## Introduction

Blepharoptosis is defined as abnormally drooping of the upper eyelid in the primary gaze, resulting in narrowing of the palpebral fissure and increasing the area of the eyelid in contact with the ocular surface. Besides the cosmetic problems involved in eyelid appearance, another direct impact of blepharoptosis could be the altered pressure exerted by the dropped eyelid and that consequently may change the underneath corneal shape^1, 2^. Using corneal topography, previous study has demonstrated significantly increased corneal asymmetry and corneal irregularity in ptotic eyes^3^. However, the corneal topography study only yields the measurement of the anterior corneal surface, it cannot reflect the alteration of the entire corneal architecture.

Recently, advances in corneal imaging techniques have allowed us to evaluate the anatomical and geometric characterization of the corneal architecture^4^. The Scheimpflug photography-based system with the capability to measure both the anterior and posterior corneal surfaces utilized a rotating Scheimpflug camera, and could provide more accurate details in corneal morphologic characteristics, including corneal elevation maps, corneal thickness spatial profile, and the percentage of thickness increase^5–7^. It has been proposed that corneal Scheimpflug tomography could help in the detection of keratoconus and topographically normal keratoconus (also called as subclinical keratoconus or forme frusta keratoconus)^5, 8–10^.

To the best of our knowledge, changes of corneal tomography in congenital blepharoptosis have not been studied in detail or reported in the literature. In this study, we aim to investigate the changes in corneal curvature, elevation, as well as pachymetric parameters over a range of severity of blepharoptosis and normal corneas by using a rotating Scheimpflug corneal tomographer, with a view to contributing to our understanding of the specific corneal structural alterations in congenital blepharoptosis.

## Results

A total of 73 eyes from 64 subjects with congenital blepharoptosis and 64 eyes from 64 normal subjects were examined. There were 35 (54.7%) patients with unilateral ptosis and 29 (45.3%) patients with bilateral ptosis. According to MRD, 22 eyes from blepharoptosis cases were divided into mild group, 25 eyes into moderate group, and 26 eyes into severe group. The characteristics of study subjects are presented in Table 1, there were no age- or sex-related statistical differences between patients with blepharoptosis and control subjects. The averages of palpebral fissure, MRD, and levator function were all significantly decreased in each blepharoptosis subgroups (*P* < 0.001, *P* < 0.001, *P* = 0.0028 in mild; all *P* < 0.001 in moderate; all *P* < 0.001 in severe, respectively). The results of refractive error are presented in Supplementary Table 1. Differences of mean refractive error between groups indicate relative hyperopic shift and increased astigmatism in blepharoptosis patients (Supplementary Table 2).

**Table 1:** Comparison of Baseline Characteristics Between Control and Blepharoptosis Groups.

	Control	Blepharoptosis		
		*Mild*	*Moderate*	*Severe*
Age (yrs)	15.73 ± 5.33	18.23 ± 5.74	14.60 ± 7.97	16.04 ± 6.93
Male (n, %)	40 (62.5)	13 (59.1)	16 (64.0)	19 (73.1)
Right eye (n,%)	35 (54.7)	13 (59.1)	13 (52.0)	12 (46.2)
Palpebral fissure (mm)	9.14 ± 0.99	6.84 ± 0.62***	5.36 ± 0.64***	3.54 ± 1.14***
MRD (mm)	4.12 ± 0.78	1.73 ± 0.37***	0.76 ± 0.26***	−1.27 ± 0.92***
Levator function (mm)	13.07 ± 1.64	10.32 ± 1.46**	5.12 ± 1.51***	2.31 ± 1.16***

mm = millimeter; MRD = marginal reflex distance.

Data are mean standard deviation unless otherwise indicated.

**P < 0.01, ***P < 0.001, versus controls.

Table 2 provides the keratometric parameters in the blepharoptosis and control groups. There were no significant differences among the four groups in terms of front K1, front K2, front J0, front J45, back K1, back K2, back J0, back J45, and Kmax. However, I-S at 4 mm and 6 mm radius rings were significantly higher in moderate (*P* = 0.007 and *P* < 0.001) and severe (all *P* < 0.001) blepharoptosis (Table 2).

**Table 2:** Comparison of Keratometric Parameters Between Control and Blepharoptosis Groups.

	Control	Blepharoptosis		
		*Mild*	*Moderate*	*Severe*
Front K1 (D)	42.23 ± 1.03	42.25 ± 1.17	42.06 ± 1.51	42.42 ± 2.18
Front K2 (D)	43.15 ± 1.14	43.29 ± 1.44	42.96 ± 1.57	43.62 ± 2.24
Front Astig (D)	0.93 ± 0.43	1.05 ± 0.50	0.90 ± 0.49	1.19 ± 0.57
Front J0 (D)	−0.051 ± 0.362	−0.116 ± 0.433	−0.115 ± 0.331	−0.102 ± 0.394
Front J45 (D)	0.046 ± 0.358	−0.011 ± 0.386	0.153 ± 0.351	−0.048 ± 0.531
Back K1 (D)	−6.05 ± 0.19	−6.05 ± 0.19	−6.02 ± 0.26	−6.10 ± 0.35
Back K2 (D)	−6.35 ± 0.22	−6.39 ± 0.27	−6.30 ± 0.30	−6.42 ± 0.42
Back Astig (D)	0.30 ± 0.12	0.32 ± 0.15	0.28 ± 0.15	0.33 ± 0.19
Back J0 (D)	0.008 ± 0.117	0.032 ± 0.145	0.038 ± 0.127	−0.006 ± 0.100
Back J45 (D)	0.018 ± 0.115	−0.001 ± 0.100	0.002 ± 0.096	0.007 ± 0.167
Kmax (D)	43.77 ± 1.20	43.98 ± 1.44	43.76 ± 1.60	44.53 ± 2.28
I-S 4 mm (D)	−0.05 ± 0.51	−0.40 ± 0.91	0.68 ± 1.39**	0.89 ± 1.41***
I-S 6 mm (D)	0.15 ± 0.67	0.18 ± 1.06	1.51 ± 1.54***	1.74 ± 1.45***

Astig = astigmatism magnitude; D = diopter; Kmax = maximum keratometry; mm = millimeter. I-S 4 mm = inferosuperior asymmetry at 4 mm radius ring; I-S 6 mm = inferosuperior asymmetry at 6 mm radius ring.

Data are mean standard deviation unless otherwise indicated.

**P < 0.01 and ***P < 0.001, versus control.

For corneal topometric indices, ISV was significantly increased in mild (*P* = 0.026), moderate (*P* < 0.001), and severe (*P* < 0.001) blepharoptosis. Parameters including IVA, KI, CKI, and IHD were also significantly increased in moderate (all *P* < 0.001) and severe (all *P* < 0.001) blepharoptosis compared to that of controls (Table 3). Significant changes of IHA were noted in patients with mild and severe blepharoptosis versus controls (*P* = 0.021 and *P* < 0.001, respectively; Table 3).

**Table 3:** Comparison of Corneal Topometric Indices Between Control and Blepharoptosis.

	Control	Blepharoptosis		
		*Mild*	*Moderate*	*Severe*
Index of surface variance	15.52 ± 3.46	19.09 ± 4.99*	21.64 ± 7.18***	25.04 ± 7.57***
Index of vertical asymmetry	0.10 ± 0.04	0.14 ± 0.05	0.19 ± 0.12***	0.22 ± 0.11***
Keratoconus index	1.02 ± 0.02	1.03 ± 0.03	1.05 ± 0.03***	1.06 ± 0.04***
Central keratoconus index	1.005 ± 0.005	1.007 ± 0.008	1.011 ± 0.008***	1.013 ± 0.007***
Index of height asymmetry	4.28 ± 3.58	7.05 ± 4.03*	4.94 ± 4.32	8.024 ± 5.16***
Index of height decentration	0.009 ± 0.004	0.012 ± 0.006	0.017 ± 0.012***	0.017 ± 0.011***

Data are mean standard deviation unless otherwise indicated.

*P < 0.05, **P < 0.01, and ***P < 0.001, versus controls.

In the elevation maps, no statistically significantly difference of variables in elevation map was noted between mild blepharoptosis and controls, except for variable of back elevation at thinnest point (*P* = 0.009) (Table 4). Parameters including front elevation at apex point, front elevation at thinnest point, maximum front elevation within central 4.0 mm zone, back elevation at apex point, back elevation at thinnest point, and maximum back elevation within central 4.0 mm zone were all significantly greater in moderate (*P* < 0.001, *P* < 0.001, *P* = 0.005, *P* < 0.001, *P* < 0.001, and *P* = 0.004, respectively) and severe (all *P* < 0.001) blepharoptosis groups than those of normal controls, whereas front or back BFS diameter did not differ significantly (Table 4).

**Table 4:** Comparison of Corneal Elevation Parameters Between Control and Blepharoptosis.

	Control	Blepharoptosis		
		*Mild*	*Moderate*	*Severe*
Elev front apex (μm)	1.86 ± 0.85	2.36 ± 1.29	2.92 ± 1.26***	3.50 ± 1.18***
Elev front thinnest (μm)	2.14 ± 1.10	2.27 ± 1.24	3.52 ± 2.29**	4.15 ± 2.89***
Elev front max 4.0 mm (μm)	3.91 ± 1.43	4.50 ± 1.66	5.24 ± 1.88**	5.92 ± 2.42***
Elev back apex (μm)	1.59 ± 2.17	2.86 ± 2.21	4.08 ± 2.63***	4.46 ± 3.39***
Elev back thinnest (μm)	3.86 ± 2.60	6.55 ± 3.61**	9.08 ± 3.46***	12.69 ± 5.38***
Elev back max 4.0 mm (μm)	10.86 ± 3.99	12.86 ± 4.37	14.44 ± 5.14**	17.62 ± 5.82***

Elev = elevated; Max = maximum; mm = millimeter; μm = micrometer.

Data are mean standard deviation unless otherwise indicated.

*P < 0.05, **P < 0.01, and ***P < 0.001, versus controls.

As illustrated in Table 5, the corneal thickness at the apex point or thinnest point in the eyes of blepharoptosis did not differ from controls. However, differences of thickness between these two points were significantly larger in mild (*P* = 0.028), moderate (*P* < 0.001), and severe (*P* < 0.001) blepharoptosis compared to that of controls. The locations of the thinnest point were also significantly changed both in mild, moderate, and severe groups (*P* = 0.026, *P* < 0.001, and *P* < 0.001, respectively). In addition, significant increases of maximum PI, average PI, maximum ART, and average ART were observed in severe blepharoptosis (*P* < 0.001, *P* = 0.001, *P* < 0.001, and *P* = 0.01, respectively).

**Table 5:** Comparison of Corneal Pharcymetric Parameters Between Control and Blepharoptosis.

	Control	Blepharoptosis		
		*Mild*	*Moderate*	*Severe*
Apex thickness (μm)	550.4 ± 31.8	559.3 ± 27.4	551.9 ± 28.9	555.8 ± 24.9
Thinnest thickness (μm)	546.5 ± 31.4	553.2 ± 28.0	544.5 ± 28.5	544.0 ± 24.7
Apex/Thinnest difference (μm)	3.70 ± 2.05	6.05 ± 3.68*	7.44 ± 4.29***	11.77 ± 5.44***
Thinnest location (mm)	0.64 ± 0.19	0.79 ± 0.25*	0.87 ± 0.23***	1.06 ± 0.29***
Max PI	1.20 ± 0.16	1.34 ± 0.19*	1.31 ± 0.26	1.52 ± 0.28***
Avg PI	0.98 ± 0.12	1.02 ± 0.12	1.01 ± 0.17	1.10 ± 0.16**
Max ART (μm)	463.8 ± 74.1	425.2 ± 83.0	428.3 ± 81.5	372.0 ± 82.5***
Avg ART (μm)	564.2 ± 77.2	552.0 ± 86.1	554.9 ± 96.2	506.0 ± 87.2*

ART = Ambrósio’s relational thickness; Avg = average; Max = maximum; mm = millimeter; PI = progression index; μm = micrometer.

Data are mean standard deviation unless otherwise indicated.

*P < 0.05, **P < 0.01 and ***P < 0.001, versus controls.

**Figure 1:**
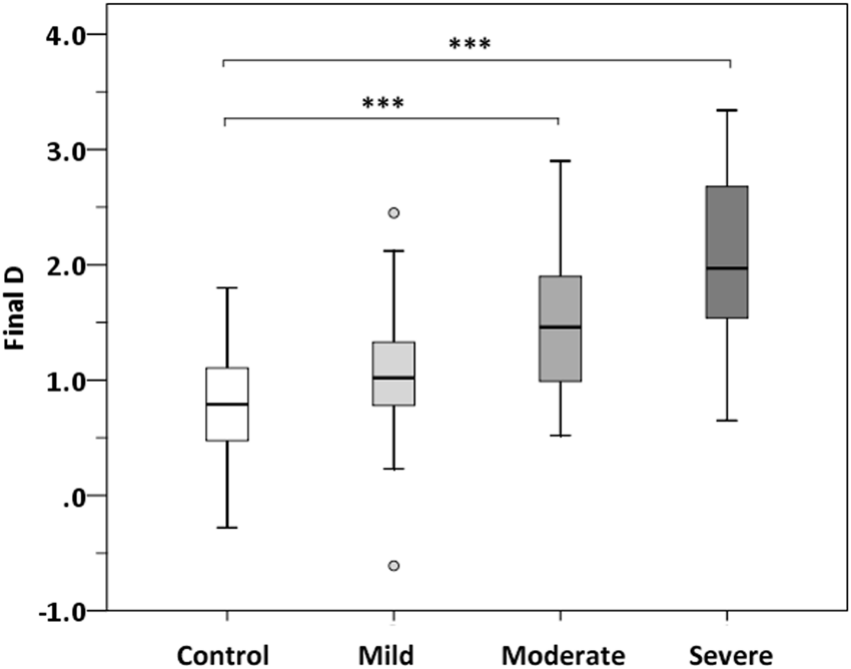
Box-and-whisker plots illustrating the distribution of final D values in healthy controls and congenital blepharoptosis groups. The final D value increased with increasing severity of blepharoptosis. ***P < 0.001, one-way ANOVA with the Dunnett’s multiple comparison test.

As shown in Fig. 1, the mean final D values were significantly higher in moderate (1.47 ± 0.57, *P* < 0.001), and severe blepharoptosis (2.03 ± 0.75, *P* < 0.001) groups than that of controls (0.75 ± 0.49). Considering a value of 2.61 as a cutoff for the final D to differentiate keratoconus from controls^11^, we found one eye (4.0%) in moderate group and nine eyes (34.6%) in severe group had final D values greater than 2.61, whereas no eye in mild blepharoptosis or normal controls had a final D value greater than 2.61.

**Figure 2:**
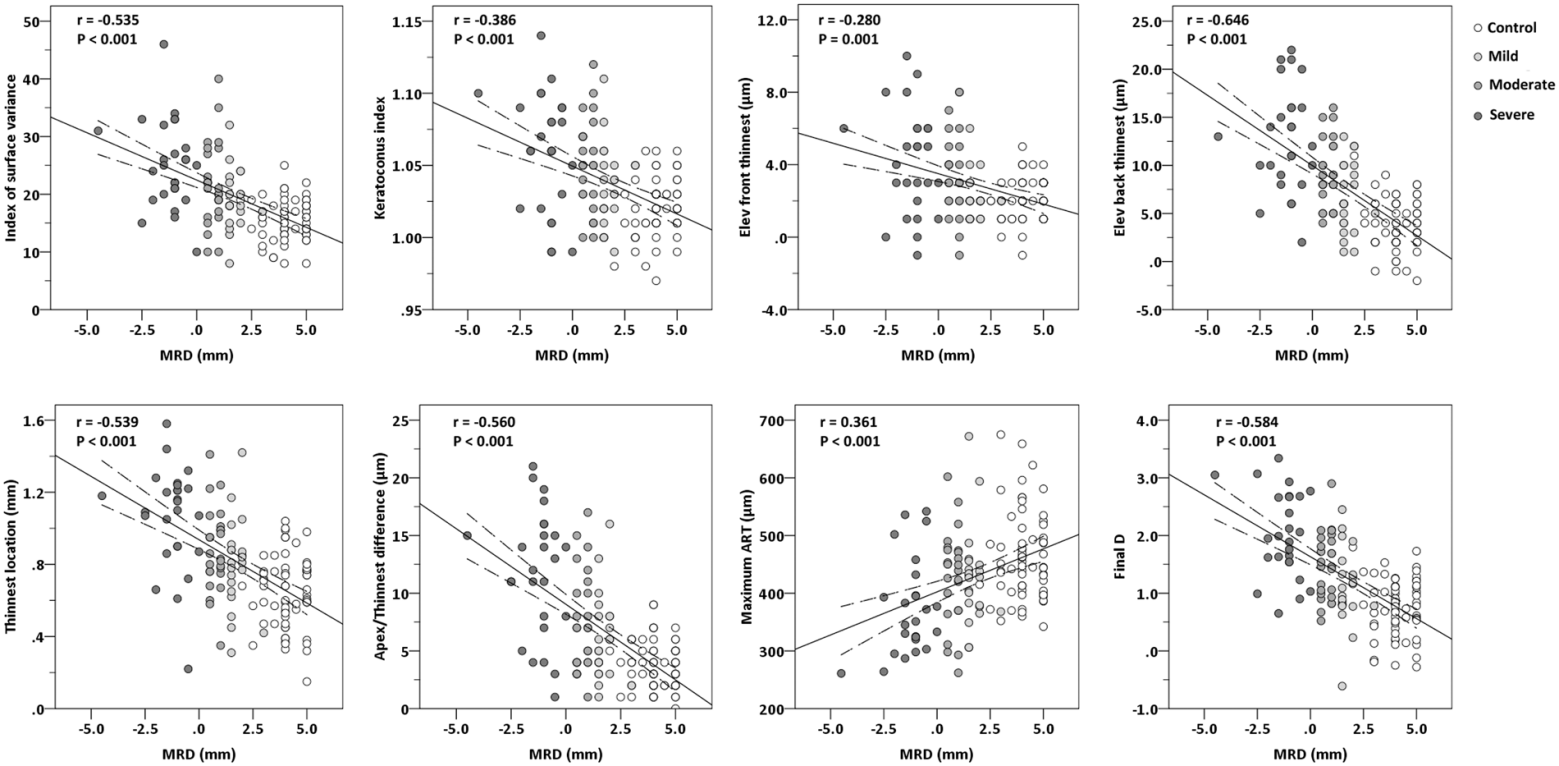
Correlations between marginal reflex distance (MRD) and corneal parameters, including index of surface variance, keratoconus index, front elevation at thinnest point, back elevation at thinnest point, thinnest location, difference between apical and thinnest thickness, maximum Ambrósio’s relational thickness (ART), and final D. Spearman rank correlation coefficients (r value) are shown with statistical significance of the correlations. The linear regression line is shown with the 95% confidence intervals of mean.

Figure 2 shows the correlations of MRD with corneal parameters. MRD were significantly and negatively correlated with ISV (r = −0.535, *P* < 0.001), KI (r = −0.386, *P* < 0.001), front elevation at thinnest point (r = −0.280, *P* = 0.001), back elevation at thinnest point (r = −0.646, *P* < 0.001), thinnest location (r = −0.539, *P* < 0.001), apex/thinnest difference (r = −0.560, *P* < 0.001), and final D (r = −0.584, *P* < 0.001). A significant positive correlation was also found between MRD and maximum ART (r = 0.361, *P* < 0.001).

**Figure 3:**
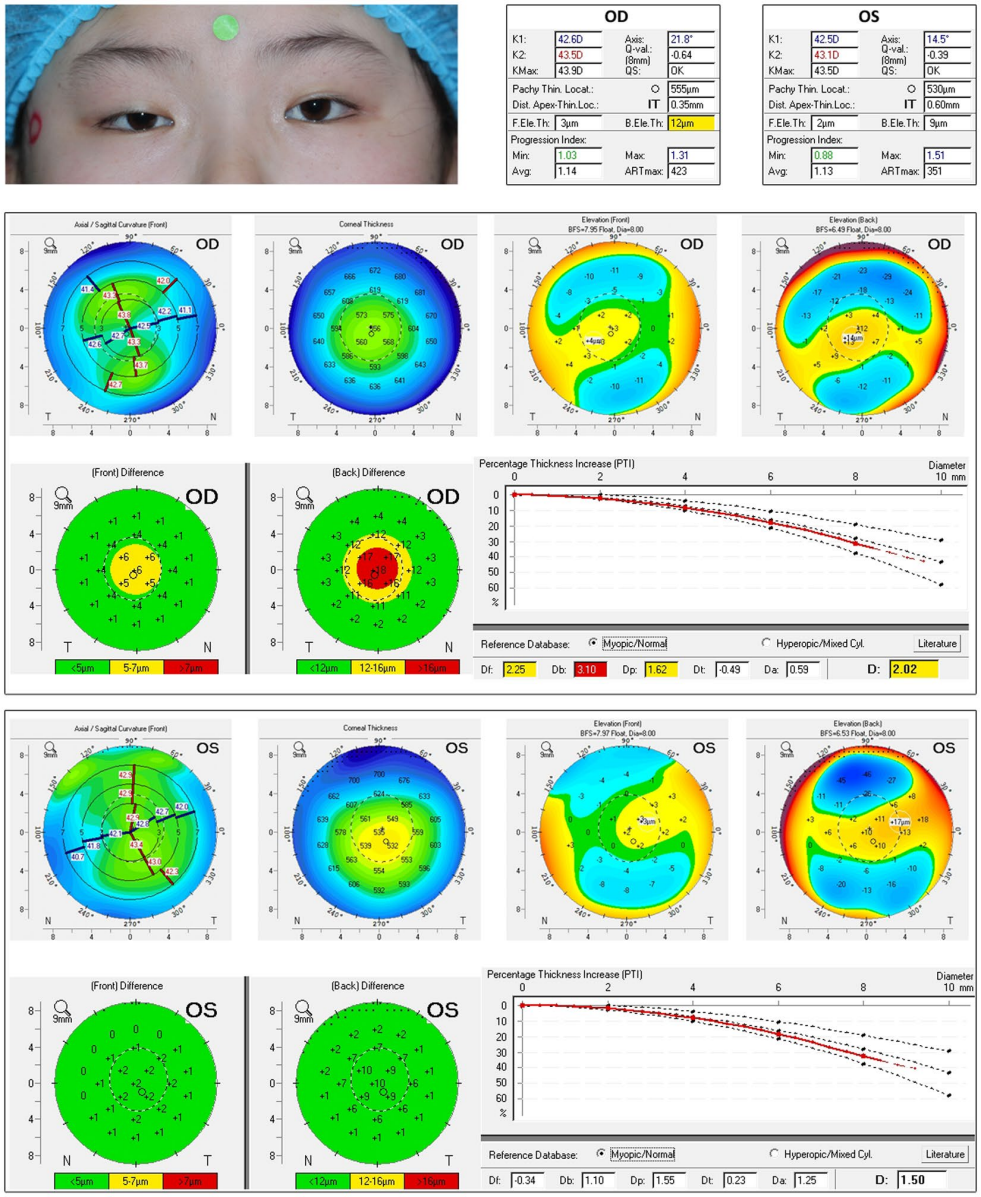
Example of a female patient included in the study with unilateral moderate blepharoptosis. In the ptotic eye (right eye [OD]), the back elevation at thinnest point shows a suspicious value of 12 μm. The elevation difference map from the anterior surface is suspicious (yellow area) with the difference value around 6 μm. The back surface is highly abnormal, with an 18-μm difference from the enhanced to standard best fit sphere. Final D index reveals a suspicious pattern, showing a value of 2.02 (yellow). The percentage thickness increase line shows no abnormality in the progression graphs. However, the nonptotic eye (left eye [OS]) shows elevation maps from front and back surfaces are within normal range.

**Figure 4:**
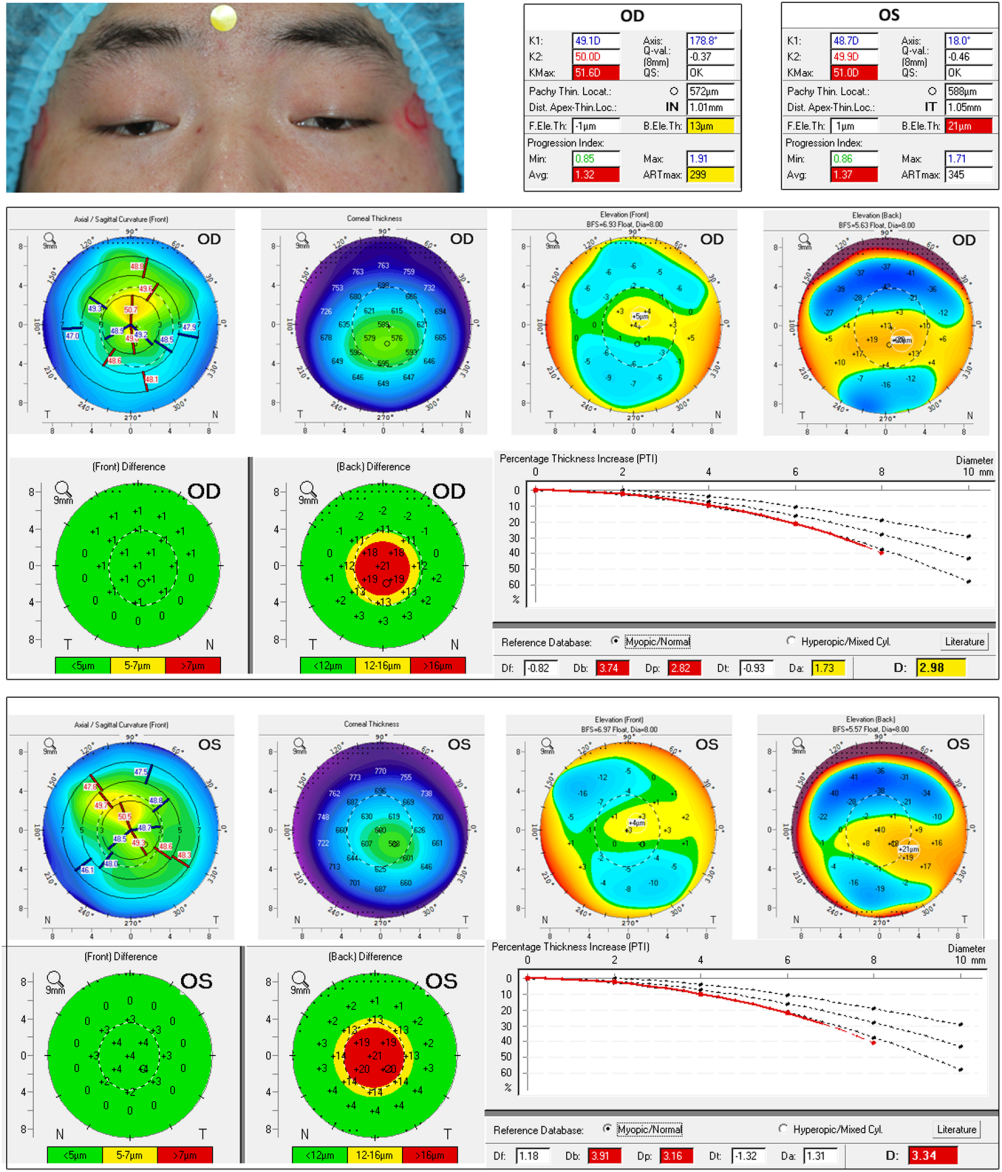
Example of a male patient included in the study with bilateral severe blepharoptosis. Both ptotic eyes show similar abnormalities. The back elevation at thinnest point is 13 μm (yellow) and 21 μm (red) in right and left eyes, respectively. Maximum Ambrósio’s relational thickness (ARTmax) is suspicious in right eye, with a value of 299 μm (yellow). The average progression index is highly abnormal in both right and left eye, with a value of 1.32 (red) and 1.37 (red), respectively. The front elevation difference maps are within normal limits, but the back surfaces in the two eyes show difference values around 21 μm. The percentage thickness increase graphs show a remarkable deviation from normality in both two eyes. The final D index is suspicious in right eye with a value of 2.98 (yellow), and clearly abnormal in left eye with a value of 3.34 (red).

Figure 3 provides a representative case of a female patient included in the study whose eyes showed unilateral moderate blepharoptosis. Elevation maps from the ptotic eye (right eye) showed abnormal findings in both anterior and posterior elevations, whereas results from the nonptotic eye (left eye) show a corneal tomography within normal limits. Figure 4 demonstrates an example of male patient with bilateral severe ptosis, he had apparently normal cornea at slit-lamp biomicroscopy, but his elevation maps and pachymetric mapping indicated early signs of corneal ectasia in both ptotic eyes, suggesting the presence of binocular subclinical keratoconus-like changes.

## Discussion

There have been relatively few reports in the literature on the effect of congenital blepharoptosis on corneal curvatures. Using corneal topography, Uğurbaş have demonstrated that the ptotic eyes had an increased incidence of corneal astigmatism, as well as higher corneal asymmetry and irregularity^3^. Although K1, K2 and astigmatism magnitude were not changed among controls and blepharoptosis groups, we found I-S at 4 mm and 6 mm radius rings were significantly increased in eyes with moderate and severe blepharoptosis. These keratometric results demonstrated no significant corneal steepening centrally but topographic asymmetry in moderate and severe blepharoptosis eyes.

In our study, most elevation parameters in both anterior and posterior corneal surfaces were all significantly increased in moderate and severe blepharoptosis eyes. It has been shown that the posterior corneal elevation at thinnest point with a cutoff value of >12 μm had 96.28% sensitivity and 98.79% specificity for discriminating keratoconus from normal eyes^11^. In the present study, the average posterior corneal elevation at thinnest point was 12.69 ± 5.38 μm in severe blepharoptosis eyes, showing remarkable increases comparing to controls. Therefore, corneas in severe congenital blepharoptosis may share similarity in elevation map with ectatic corneas of subclinical keratoconus^11, 12^.

Additionally, we found most pachymetric metrics in severe blepharoptosis group also had similar results with that in studies of subclinical keratoconus, including greater apex/thinnest difference, increased thinnest location value, increased PI, and decreased ART indices^8–10^—all these results indicated the presence of focalized corneal thinning in the eyes with severe blepharoptosis.

The final D index from the Belin/Ambrósio Enhanced Ectasia Display is a multimetric combination parameter composed of keratometric, pachymetric, pachymetric progression, and back elevation parameters. It is suggested that the final D index could be used as the sole parameter to identify early corneal ectasia^10^. Using final D value greater than 2.61 may help to identify majority of keratoconus suspects truly have disease^13^. In the present study, there were 9 out of 23 eyes in blepharoptosis group had final D values greater than 2.61, and that indicated a high risk of subclinical keratoconus-like changes occurs in severe blepharoptosis eyes.

Although we observed a high proportion of eyes with subclinical keratoconus-like changes in severe blepharoptosis group, congenital blepharoptosis associated with overt keratoconus was rarely seen in our routine clinic work or reported in literature. It is reasonable that corneal alterations in congenital blepharoptosis may be confined to subclinical keratoconus-like changes. As known, the etiology of keratoconus remains elusive, but is multifactorial combining with genetic and environmental factors^14^. A common belief is that environmental factors (eye rubbing) may trigger the disease in genetically susceptible patients^14, 15^. Thus, a single factor of lid compressive force alone may hardly induce clinical keratoconus in simple congenital blepharoptosis. Additionally, mechanical force from dropped upper eyelid seems to be much weaker compared to vigorous eye rubbing. Sakai *et al*.^16^ reported a mean upper eyelid pressure of 16.95 ± 6.08 mm Hg by using a tactile pressure sensor, whereas eye rubbing in patients with keratoconus used their knuckles could generate a force of >4.5 kg/2.54 cm^2^ (approximately 1,300 mm Hg)^17^.

Several limitations of this study need to be considered. First, all included subjects were Chinese. Since the anatomy of eyelids and orbits were different between Asian and Western populations, the accurate definition and classification of blepharoptosis also differ among these two groups^18, 19^. Whether similar results could be observed in other races needs further studies. A second limitation of our study is the small sample size, because we need well-controlled and well cooperated participants. Third, this study was conducted in our eye center, which may prone our results to a hospital-based bias. At last, it is important to be aware of the predictive limitations of our cross-sectional study. Although the cross-sectional design allowed us to provide evidence of an association between congenital blepharoptosis and subclinical keratoconus-like changes, longitudinal design studies are necessary to establish a true cause and effect relationship.

In summary, we confirmed that congenital blepharoptosis not only induced corneal asymmetry and irregularity, but also affected corneal tomography, such as increased corneal elevation in blepharoptosis with more than moderate severity and even focalized corneal thinning in severe cases.

## Methods

### Subjects and Clinical Evaluations

This prospective, case-control study included patients with congenital blepharoptosis, and candidates for refractive surgery or orthokeratology contact lens with normal corneas. Only emmetropic and myopic (range −0.50 to −10.00 diopters) patients with normal eyelid position were considered as normal controls in this study^20^. All participants were enrolled among consecutive patients examined at the Eye Center of Second Affiliated Hospital, Medical College of Zhejiang University, from November 2015 through June 2016. Control subjects were enrolled to match the age and sex distribution of the disease group. Ophthalmic examinations consisted of best-corrected visual acuity measurements, slit-lamp examination, cover test, and extraocular movements. The retinoscopic refraction under cycloplegia was used for further analysis. The palpebral fissure, upper eyelid marginal reflex distance (MRD), levator function, and Bell’s phenomenon were also evaluated twice by a senior doctor (JY). MRD was measured in primary gaze with the frontalis muscle fixed, and was recorded in increments of 0.5 mm using a metric ruler. Congenital blepharoptosis was diagnosed and verified by parental history or a photograph captured within the first few months of life. We defined blepharoptosis as the presentation of a MRD of <2 mm as suggested by previous study^18^, and/or asymmetry between both upper eyelids height was ≥1 mm in unilateral cases^19^. According to MRD, the severity of ptosis was classified into three grades: mild (MRD >1 mm), moderate (0 mm <MRD ≤1 mm), and severe (MRD ≤0 mm). In the case of bilateral ptosis with equal severity, only one eye was randomly chosen for the study. Patients with the following conditions were excluded from the study: associated syndromes, such as Horner syndrome, congenital third cranial nerve palsy; blepharophimosis, or Marcus Gunn jaw-winking syndrome; strabismus; nystagmus; poor Bell’s phenomenon; history of ocular or eyelid surgery; significant hyperopia (> +1 diopter); corneal abnormalities due to other factors such as trauma, keratoconus, chronic eye rubbing, and vernal keratoconjunctivitis; and anyone not able to cooperate with examinations.

The participants who wore rigid contact lenses were asked to stop using them for 5 weeks, and the use of soft contact lenses was stopped for at least 2 weeks before this assessment.

All patients or their parents gave informed consent to participate in this study. Patients in Figs 3 and 4 also provided written informed consent for the publication of their eye photos. The research protocol followed the tenets of the Declaration of Helsinki and was approved by the ethics committee of the Second Affiliated Hospital, Zhejiang University School of Medicine.

### Corneal Tomography

The Pentacam HR system (Oculus, Wetzlar, Germany) was used to evaluate the anterior and posterior corneal surfaces. The measurements were performed in the automatic release mode by the same experienced examiner, and 25 Scheimpflug images were obtained for each eye within 2 seconds. In the situation of blepharoptosis, the ptotic eyelid was gently lifted by the examiner without any extra pressure on the eyeball before image acquisition. Only measurements with an “OK” reading in the quality specification window were accepted for further analysis, otherwise scans were repeated. Two qualified measurements were averaged for statistical analysis. The sagittal curvature, front elevation, back elevation, corneal thickness, and Belin/Ambrósio Enhanced Ectasia Display were evaluated. Elevation data were measured in standardized fashion relative to a reference best-fit sphere (BFS) calculated at a fixed optical zone of 8.0 mm.

The following data were obtained from Pentacam system: (1) keratometric values: flat keratometry (K1), steep keratometry (K2), astigmatism altitude and axis for the central 3.0 mm of cornea, maximum keratometry (Kmax), and anterior inferosuperior asymmetry (I-S) at 4 and 6 mm radius rings from the cornea apex; (2) topometric indices: index of surface variance (ISV), index of vertical asymmetry (IVA), keratoconus index (KI), center keratoconus index (CKI), index of height asymmetry (IHA), index of height decentration (IHD); (3) variables in elevation map: elevation at apex point, elevation at thinnest point, and maximum elevation within central 4.0 mm zone; (4) corneal pachymetric parameters: corneal thickness at the apex and at the thinnest point, difference of thickness between these two points (apex/thinnest difference), thinnest location, pachymetric progression indices (PI) and Ambrósio’s relational thickness (ART); and (5) final D value.

When analyzing the astigmatisms of both corneal surfaces, the astigmatism value was converted to the rectangular forms of Fourier notation (J0 [Jackson cross-cylinder with axes at 180° and 90°] and J45 [Jackson cross-cylinder with axes at 45° and 135°]) for the power vector analysis using the following equations: J0 = (−C/2) cos 2α and J45 = (−C/2) sin 2α, where C was the corneal astigmatism magnitude, and α was the meridian of steep keratometry^21^.

### Statistical Analysis

One eye of each normal subject was randomly chosen for data analysis. Where patients had bilateral blepharoptosis with symmetric grade, one eye was also chosen at random for the analysis. Refractive data was analyzed by using dioptric power matrices as described by Kaye and Harris^22^. Normal distribution of data was confirmed with the Shapiro-Wilk test. A multivariate generalized linear model had been used before analyzing the corneal tomography indices between groups, Dunnett’s test was chosen for post hoc multiple comparisons. Spearman correlation analyses were used to define the correlation between MRD and Pentacam parameters. All statistical analyses were performed using the Statistical Package for the Social Science software version 20.0 (SPSS Inc., Chicago, IL). A *P* value < 0.05 was considered statistically significant.

## Electronic supplementary material

Supplementary Information
