# Supplementary material for: Changes of corneal tomography in patients with congenital blepharoptosis
Source: Sci Rep. 2017 Jul 26;7:6580. doi: 10.1038/s41598-017-06823-7 (PMC5529457; doi:10.1038/s41598-017-06823-7)
Supplement: Supplementary file 1 — Supplementary Information [file 41598_2017_6823_MOESM1_ESM.pdf]

Changes of corneal tomography in patients with congenital blepharoptosis

Tiepei Zhu MD, Xin Ye MD, Peifang Xu MS, Jingyi Wang MS, Huina Zhang PhD, Hailong Ni MD, Zhaoan Su MD, Juan Ye MD

Supplementary Table 1. Mean refractive error in control and blepharoptosis groups

	**Standard Notation**			**Matrix Components**		
	Sphere (D)	Cylinder (D)	Axis	f_{11} (D)	f_{12} (D)	f_{22} (D)
Control	-3.45	+0.36	82	-3.09	-0.05	-3.44
Mild	-3.20	+0.46	82	-2.75	-0.07	-3.19
Moderate	-1.78	+0.60	88	-1.17	-0.02	-1.78
Severe	-2.33	+0.97	81	-1.39	-0.15	-2.31

Supplementary Table 2. Difference of mean refractive error between control and blepharoptosis groups

	Standard Notation			Matrix Components		
	Sphere (D)	Cylinder (D)	Axis	Δf_{11} (D)	Δf_{12} (D)	Δf_{22} (D)
Mild-Control	+0.25	+0.1	79	+0.35	-0.02	+0.25
Moderate-Control	+1.66	+0.26	96	+1.92	+0.03	+1.66
Severe-Control	+1.11	+0.61	80	+1.70	-0.10	+1.13
